# Seasonal succession of microbial community co-occurrence patterns and community assembly mechanism in coal mining subsidence lakes

**DOI:** 10.3389/fmicb.2023.1098236

**Published:** 2023-02-03

**Authors:** Wangkai Fang, Tingyu Fan, Liangji Xu, Shun Wang, Xingming Wang, Akang Lu, Yongchun Chen

**Affiliations:** ^1^School of Earth and Environment, Anhui University of Science and Technology, Huainan, China; ^2^Anhui Engineering Laboratory for Comprehensive Utilization of Water and Soil Resources and Ecological Protection in Mining Area With High Groundwater Level, Huainan, China; ^3^National Engineering Laboratory of Coal Mine Ecological Environment Protection, Huainan, China

**Keywords:** subsidence lake, seasonal succession, bacterial community diversity, co-occurrence network, keystone species, community assembly

## Abstract

Coal mining subsidence lakes are classic hydrologic characteristics created by underground coal mining and represent severe anthropogenic disturbances and environmental challenges. However, the assembly mechanisms and diversity of microbial communities shaped by such environments are poorly understood yet. In this study, we explored aquatic bacterial community diversity and ecological assembly processes in subsidence lakes during winter and summer using 16S rRNA gene sequencing. We observed that clear bacterial community structure was driven by seasonality more than by habitat, and the α-diversity and functional diversity of the bacterial community in summer were significantly higher than in winter (*p* < 0.001). Canonical correspondence analysis indicated that temperature and chlorophyll-a were the most crucial contributing factors influencing the community season variations in subsidence lakes. Specifically, temperature and chlorophyll-a explained 18.26 and 14.69% of the community season variation, respectively. The bacterial community variation was driven by deterministic processes in winter but dominated by stochastic processes in summer. Compared to winter, the network of bacterial communities in summer exhibited a higher average degree, modularity, and keystone taxa (hubs and connectors in a network), thereby forming a highly complex and stable community structure. These results illustrate the clear season heterogeneity of bacterial communities in subsidence lakes and provide new insights into revealing the effects of seasonal succession on microbial assembly processes in coal mining subsidence lake ecosystems.

## Introduction

As one of the important energy sources in the world, coal plays a crucial role in economic development (Liu et al., 2018). Currently, underground coal mining accounts for a larger ratio than open cut mining in global coal production (Mason et al., 2021). The large-scale and high-intensity exploitation of coal resources has promoted the rapid development of the world economy (Sun et al., 2020). However, this process inevitably causes grave ecological and environmental problems (Topp et al., 2010). One of the main problems is land collapse owing to underground coal mining (Li et al., 2022). For example, every 10,000 tons of underground coal mining in China will lead to approximately 0.002–0.0033 km^2^ of land subsidence (Xu et al., 2022). By the end of 2017, the subsidence area created by underground coal mining exceeded 20,000 km^2^, with a growth rate of approximately 700 km^2^ per year in China (Chen et al., 2020). With the impact of high groundwater levels, rainfall, and surface runoff, the large-scale subsidence area formed by excessive underground coal mining transforms the original terrestrial ecosystem into a water–land complex ecosystem, and the soil of coal mining subsidence changes into the sediment of the aquatic environment (Ouyang et al., 2018). Additionally, some subsidence water bodies impacted by sustained underground coal mining extends the range of subsidence and increasingly connect with natural waters, eventually forming an open subsidence lake (Fan et al., 2022). As the most varied and abundant groups of biota on Earth (Shoemaker et al., 2017), microbes in aquatic and terrestrial ecosystems can quickly respond to anthropogenic disturbances and climate changes and perform a crucial role in a variety of ecological functions, including nutrient cycling, pollutant degradation, and regulating energy flow (Hahn, 2006; Delgado-Baquerizo et al., 2016; Crowther et al., 2020).

According to statistics, 70% of the key coal mining areas in China belong to water shortage areas, and 40% belong to severe water shortage (Pan et al., 2012). Coal mining subsidence lakes have gradually become a new source of surface water in the coal mining area, which has largely alleviated the problem of water shortage around the mining area (Hu et al., 2021). Based on the different utilization modes of subsidence lakes, different types of water bodies, such as aquaculture waters, wetlands, and plain reservoirs, have been formed, which perform crucial work in sustaining ecological balance (Yi et al., 2014). Research on subsidence lakes in recent years has mainly concentrated on the distribution characteristics of heavy metals (An et al., 2020; Chen et al., 2022), nutrients (Chen et al., 2020; Zha et al., 2021), polycyclic aromatic hydrocarbons (Ouyang et al., 2018), and the evaluation of lake ecosystem service value (Demirbugan, 2019; Xu et al., 2020). At present, the knowledge gap about the temporal and spatial succession characteristics of microbial communities in subsidence lakes still exists. How and to what extent climate change affects the microbial community structure and community assembly process in subsidence lakes has not been fully explored. This limits our comprehension of the structure and function in the subsidence lake ecosystem and hinders the effective utilization of water resources in mining areas.

Determining the mechanisms and processes of microbial diversity in reply to climate variation is a primary topic in microbial ecology (Nemergut et al., 2013; Zhou et al., 2014). Deterministic and stochastic processes compose two crucial and complementary mechanisms in the comprehension of microbial community assembly processes (Hubbell, 2005; Sloan et al., 2006). Deterministic processes refer to nonrandom and the mechanisms based on niche involving environmental filtering and interspecific interactions (promotion, competition, reciprocity, and predation; Stegen et al., 2012). These factors form microbial environmental fitness and largely decide the composition and abundance of microbial (Tang et al., 2020; Wan et al., 2021). In contrast, the neutral theory emphasizes that the stochastic process mainly controls the microbial community assembly, including ecological drift, random speciation, death, and dispersal events (Chase and Myers, 2011). It is widely accepted that microbial community variation is controlled by the processes deterministic and stochastic simultaneously, whereas the significance of deterministic or stochastic processes could alter across ecosystems and seasons (Vellend et al., 2014; Zhou et al., 2022b). Therefore, environmental changes such as global warming may affect microbial assembly through regulating deterministic and stochastic processes (Zhu et al., 2020). Microbial community assembly mechanisms have been well explored in urban rivers (Zhang et al., 2019b; Niu et al., 2021), eutrophic lakes (Tang et al., 2021; Wan et al., 2021), and soil ecosystems (He et al., 2022; Wang et al., 2022a). The impact of environmental changes on microbial community assembly and interspecific interactions in subsidence lakes is still unclear.

In this study, we used the coal mining subsidence lake in Huainan as a model ecosystem to explore the seasonal variation in bacterial community structure and community assembly mechanism in the subsidence lake based on the null model and network analysis. We hypothesized that seasonal variation may perform a stronger influence on the bacterial community structure and diversity than spatial variation. Additionally, we hypothesized that the seasonal variation of bacterial communities is dominated by deterministic processes in subsidence lakes. Finally, we hypothesized that the bacterial community network in summer was more complex and stable than that in winter due to the suitable temperature and sufficient nutrition in summer. This research was the first attempt to investigate the bacterial community structure and assembly mechanism in coal mining subsidence lakes. Therefore, it will make a fundamental contribution to the management and efficient exploitation of water resources under the circumstance of the future increase in subsidence lake area and eutrophication degree in the mining area.

## Materials and methods

### Study area and sample collection

**Figure 1:**
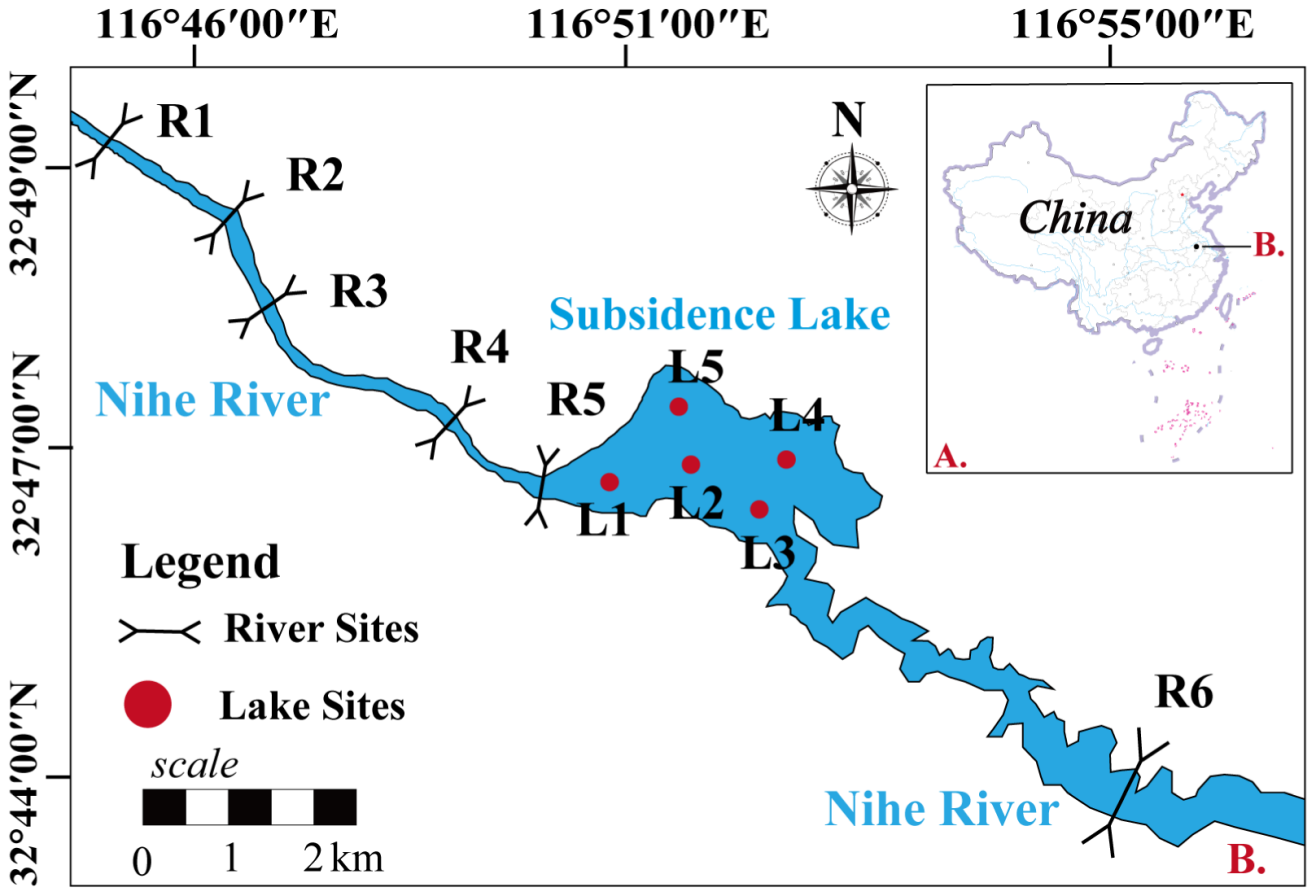
The sites of water samples obtained in the subsidence lake.

The coal mining subsidence lake (116°45′38″–116°55′7″E and 32°44′48″–32°49′54″N) is in Huainan city, Anhui Province, China (Figure 1). Subsidence lakes are created by the subsidence of underground coal mining void areas under the action of gravity, and their shape is irregular. The subsidence area continues to increase and connects with adjacent rivers (Nihe River), eventually forming an open subsidence lake. The water source of the subsidence lake mainly comes from groundwater, river water, rainfall, and surface runoff (Hou et al., 2006). The average water depth is 5.52 m, and the water area is 4.9 km^2^. The subsidence lakes with an average annual precipitation of approximately 926 mm and average annual evaporation of 1442.9 mm (He et al., 2005). Nihe River is the major river adjacent to the lake, and the subsidence lake is located in the middle reaches of the Nihe River. The heavy metal content is lower than the class III standard of China’s “Environmental quality standards for surface water” (GB3838-2002**)** in subsidence lake, and there is no heavy metal pollution (Zhang et al., 2022). The main utilization methods of subsidence lakes are fish farming and irrigation water for farmland. The pollution sources around the subsidence lakes are mainly domestic and agricultural wastewater, and the eutrophication of water body is relatively serious.

To investigate the impact of natural river confluence on the subsidence lakes, five sites (R1–R5) were set up in the upper reaches of the Nihe river. Five sites (L1–L5) were set up in the subsidence lake to capture the spatial dynamics of the microbial community. One sampling site (R6) was also set up in downstream of the Nihe river to analyze the impact of water outflow from the subsidence lake on the natural river. The upper reaches of the Nihe river are mainly surrounded by villages and large areas of farmland, and agricultural and domestic pollution is more serious. The main utilization of the subsidence lake is fish farming and irrigation of farmland, which is influenced by agricultural production.

Water samples were obtained in January (winter) and June (summer) 2021. A composite water sample (three grab samples) was obtained based on a plexiglass water collector at a depth of 0.5 m below the lake surface. Samples were preserved in a pre-rinsed sterile plastic bottle at 4°C and brought to the laboratory within 12 h. In the laboratory, water samples were separated into two subsamples. The 0.22-μm pore prewashed polycarbonate filters were used to filter one subsample. The filters were collected in sterile centrifuge tubes and then stored at −80°C before extraction of DNA. Another subsample was used for physicochemical analysis immediately.

### Physiochemical monitoring

At each sampling point, a multiparameter water quality sonde (YSI 6600 V2, United States) was used to measure temperature (T), pH, oxidation–reduction potential (ORP), dissolved oxygen (DO), and electrical conductivity (EC). Total nitrogen (TN), total phosphorus (TP), nitrite (NO_2_^−^–N), ammonia nitrogen (NH_4_^+^–N), chemical oxygen demand (COD), chlorophyll-a (Chl-a), nitrate (NO_3_^−^–N), and biochemical oxygen demand (BOD) were evaluated by reference the standard methods (Jin and Tu, 1990).

### DNA extraction and PCR amplification

Based on the manufacturer’s instructions, the Fast DNA Spin Kit for Soil (MP Biomedical, United States) was used to extract the total DNA from the filters. NanoDrop 2000 (Thermo Fisher Scientific, MA, United States) was used to evaluate the concentration of DNA, and electrophoresis on a 1% agarose gel was used to assess DNA quality. The V3–V4 regions genes of bacterial 16S rRNA were amplified based on the primer sets 338F (5′-ACTCCTACGGGAGGCAGCA-3′) and 806R (5′-GGACTACHVGGGTWTCTAAT-3′). Axyprep DNA Gel extraction kit (Axygen, United States) was used to purify the PCR products. An equimolar solution was used to pool purified PCR products and paired-end-sequenced, and then sent to Personal Biotechnology Co., Ltd., (Shanghai, China) for sequencing on the MiSeq PE300 platform (Illumina, United States). Raw sequencing reads have been submitted to the National Coalition Building Institute (NCBI), with project number PRJNA864567.

### Sequence analyses

The 16S gene amplicon sequence data were handled using the DADA2 algorithm and Qiime2 pipeline (Callahan et al., 2016; Bolyen et al., 2019). After the process of quality control, merging of paired sequences, removal of chimeric sequences, the tables of amplicon sequence variant (ASV) were created. The Silva database (version 132) was used to annotate taxonomic information of the ASVs (Quast et al., 2012). The ASVs which were classified as chloroplasts and mitochondria were deleted outside of the next analysis.

### Statistical analyses

The similarity of different samples was presented using the Bray-Curtis distance in Principal coordinates analysis (PCoA). The Wilcoxon test was used to calculate the difference of physicochemical factors, community composition, and diversity in different samples. The environmental factors that lead to the variation of community and function were estimated by canonical correspondence analysis (CCA) and variation partition analysis (VPA; Liu et al., 2015). The package “rdacca.hp” was used to estimate the contribution of different environmental factors (Lai et al., 2022). Functional annotation of taxa in different samples was conducted based on the package of FAPROTAX (Louca et al., 2016). The Spearman’s correlations between dissimilarity entries of physicochemical parameters (Euclidean distance), community composition (Bray–Curtis distance), and geographic distance (Euclidean distance) were performed based on mantel permutation test, vegan and linkET packages (Legendre and Legendre, 2012).

The assembly mechanism of the microbial community in subsidence lake and natural river was estimated using the Normalized stochasticity ratio (NST; Ning et al., 2019). The value of NST < 0.5 suggests that bacterial community composition (BCC) was derived in deterministic process, while the value of NST > 0.5 suggests that BCC was derived by stochastic process. Based on Jaccard similarity metrics and the NST package, NST was calculated in R (Ning et al., 2019).

Networks were performed to compare the co-occurrence relationship of microbial communities based on Spearman’s correlation in R using the “psych” package (Yang et al., 2021). The ASVs were selected to analyze which relative abundance > 0.05% to simplify the set. When constructing networks, the correlation coefficients |r| > 0.9 and adjusted *p*-values < 0.01 was considerate (Barberán et al., 2012). The “igraph” package was used to conduct network topological properties and random networks were in R (Csardi and Nepusz, 2006). The interactive Gephi platform was used to visualize these networks (Bastian et al., 2009). Based on within-module connectivity (Zi) and among-module connectivity (Pi), the nodes of the network were classified (Guimera and Nunes Amaral, 2005). These nodes could be defined to four categories, namely peripheral nodes, connectors, module hubs, and network hubs, in which the range of Zi and Pi value was Zi < 2.5, Pi < 0.62; Zi < 2.5, Pi > 0.62; Zi > 2.5, Pi < 0.62; Zi > 2.5, Pi > 0.62, respectively. In those classifications, generally considered as keystone species were the network hubs, connectors and module hubs (Nemergut et al., 2013).

## Results

### Spatial and temporal variation in the diversity of bacterial communities

**Figure 2:**
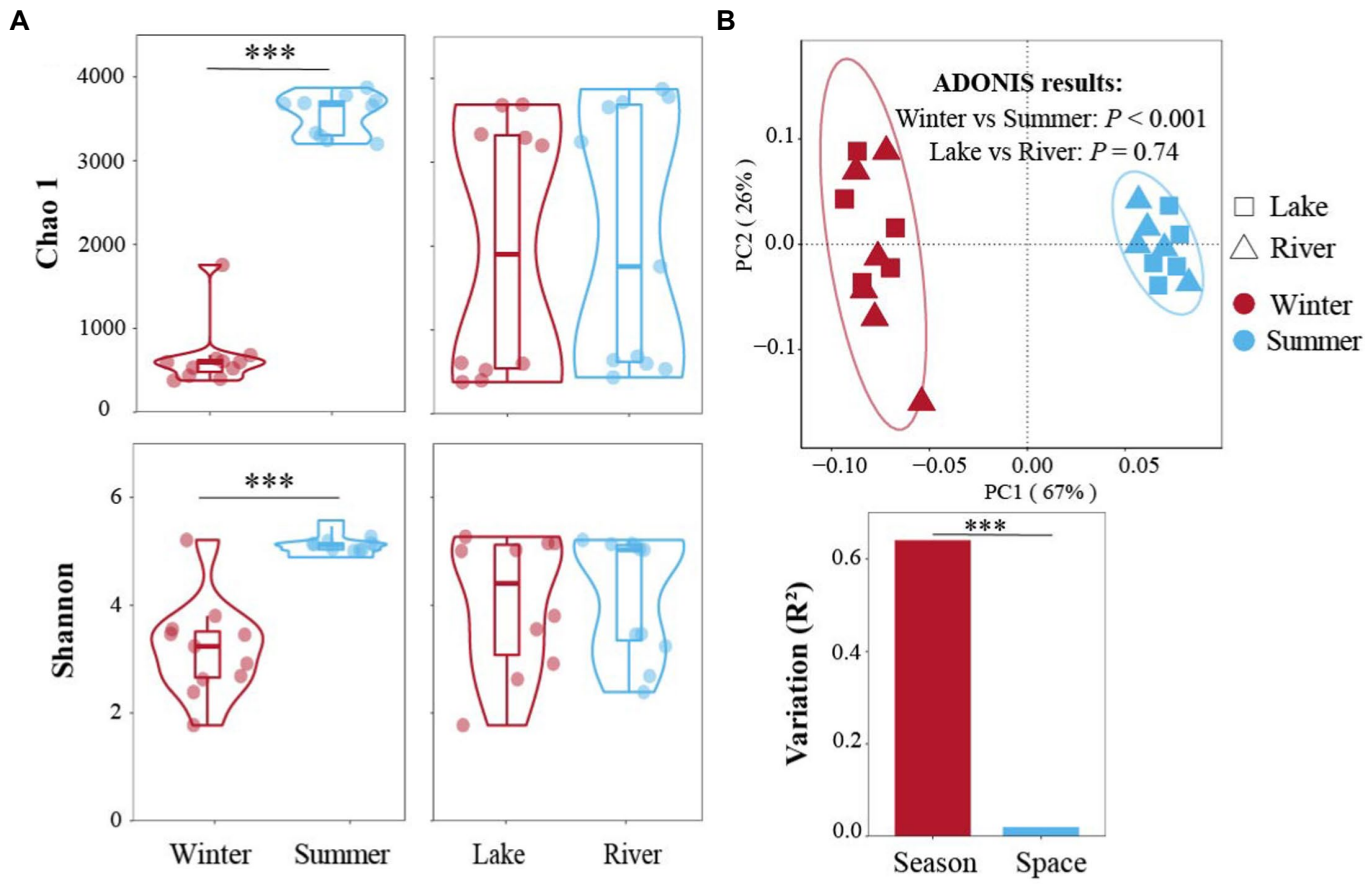
Bacterial community diversity in winter and summer across subsidence lake and natural river. **(A)** The difference of Chao 1 and Shannon α-diversity index between different seasons and sites. **(B)** Bray Curtis distances-based PCoA of bacterial community structures among different spatial and temporal scales using adonis analysis. Two comparisons with Wilcoxon tests, ^*^p < 0.05, ^**^p < 0.01, ^***^p < 0.001.

Overall, 1,779,660 high-quality reads were acquired, and 32,113 amplicon sequence variants (ASVs) were annotated from those reads in this study. The rarefaction curves approached saturation, showing sufficient sequencing depth for downstream analyses (Supplementary Figure S1). The Chao 1 and Shannon indices in summer were 3,546 and 5.1, respectively, higher than in winter significantly (648 and 3.2, respectively; Figure 2A; Wilcoxon test, *p* < 0.001). In contrast, the Chao 1 and Shannon indices not observed significant differences in the subsidence lakes (1969 and 4.1, respectively) and natural rivers (2082 and 4.2, respectively; Figure 2A; Wilcoxon test, *p* > 0.05).

Bray Curtis-based PCoA was conducted to display the β-diversity patterns of bacterial communities in different seasons and sites (Figure 2B). The PCoA illustrated that 67% of the bacterial communities variation was explained by the first axis. The adonis results demonstrated that the community structure was significantly separated from winter and summer (*p* < 0.001); whereas, there did not found significant variation between the subsidence lake and the natural river (*p* = 0.74; Figure 2B). These results showed that the seasonal impacts (*R*^2^ = 0.64) were much more distinct than the spatial effects (*R*^2^ = 0.02) in the subsidence lake and natural river (*p* < 0.001; Figure 2B).

### Structure and predicted function of bacterial community at the different seasons

**Figure 3:**
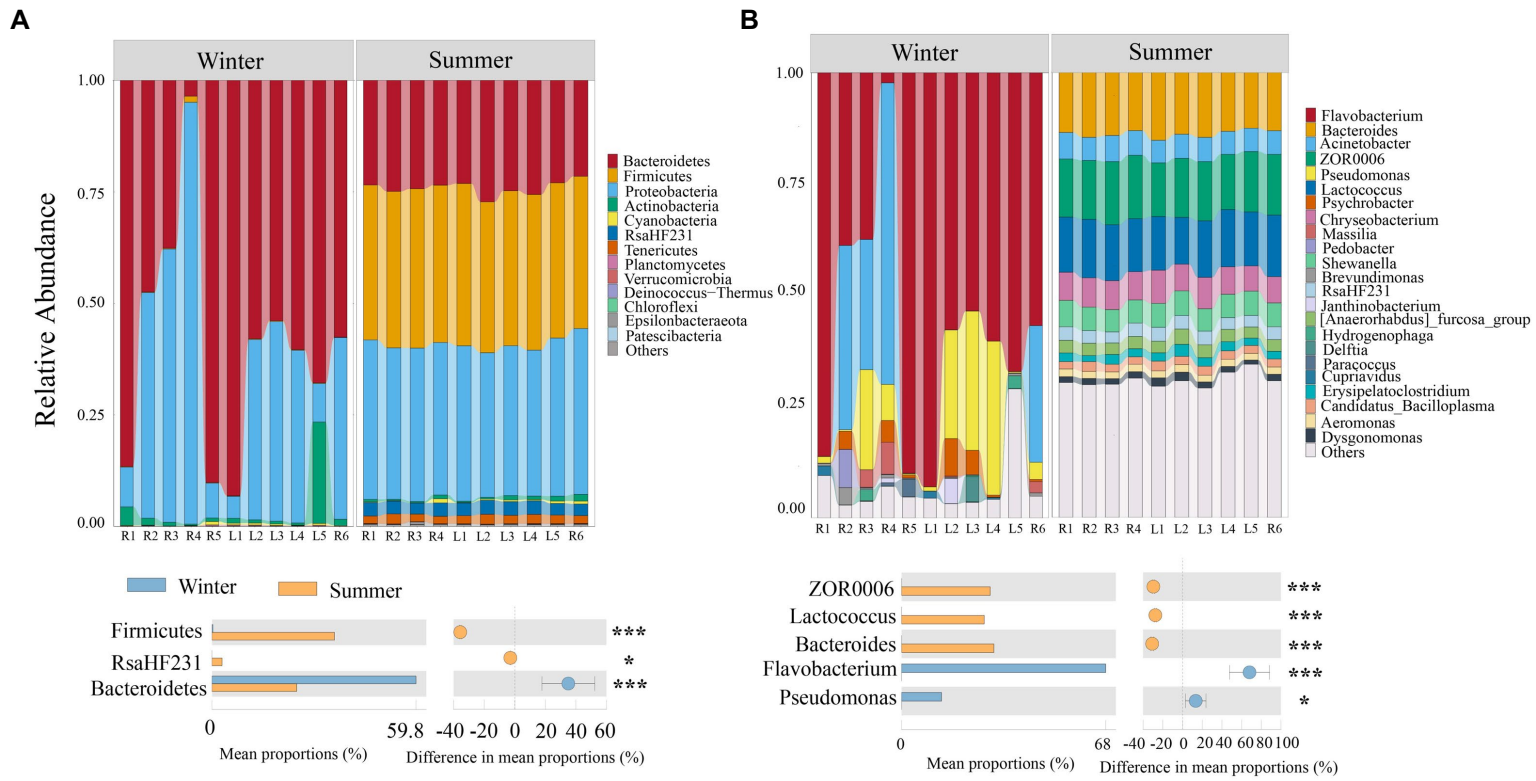
Temporal and spatial distribution of bacterial community in subsidence lake and natural river. **(A)** Distribution of bacterial communities composition at different levels. **(B)** Significantly different bacterial community compositions between different seasons. Two comparisons with Wilcoxon tests, ^*^p < 0.05, ^**^p < 0.01, ^***^p < 0.001.

The community structure of bacterial at the phylum and genus levels varied among different seasons and sites (Figure 3). Samples from winter were dominated by *Bacteroidetes* (59.6%), followed by *Proteobacteria* (36.4%), *Cyanobacteria* (0.25%) and *Actinobacteria* (3.2%); while the most dominated phyla of bacterial in summer were *Firmicutes* (35.1%), *Proteobacteria* (34.4%), *Bacteroidetes* (24.1%), *RsaHF231* (2.8%) and *Tenericutes* (1.9%; Figure 3A). At the genus level, the most dominated genus in winter was *Flavobacterium* (58.4%), *Acinetobacter* (15.4%), *Pseudomonas* (11.6%) and *Psychrobacter* (2.2%); while the most dominated genus in summer was *Bacteroides* (13.9%), *ZOR0006* (13.3%), *Lactococcus* (12.4%), *Chryseobacterium* (6.4%) and *Acinetobacter* (5.4%; Figure 3A). The dominant bacterial community in winter and summer also show significant differences (Wilcoxon test, *p* < 0.001; Figure 3B).

The predicted functional profiles of the bacterial community were further assessed using FAPROTAX (Supplementary Figure S2). A total of 47 annotated functional groups of the bacterial community were obtained in winter, 55 functional groups were founded in summer (Supplementary Figure S2), which suggests higher functional diversity in summer. These predicted functional groups involved the nitrogen cycle, sulfur cycle, carbon cycle, energy source, and other functions. Between the predicted functional, aromatic compound degradation, chemoheterotrophy, animal parasites or symbionts and human-associated were the most enriched groups in both winter and summer. Moreover, Wilcoxon test results found that the function of aerobic chemoheterotrophy and ureolysis were the abundant groups in winter (*p* < 0.001), while the functional groups related to the nitrate reduction, nitrate respiration, fermentation, and plant-pathogen were enriched in summer (*p* < 0.001; Supplementary Figure S3).

### Influence of geographic and environmental factors on bacterial community structure in subsidence lakes

Our results suggested that the bacterial community similarity and geographic factors showed slight negative correlations (*r* = −0.05, *p* = 0.733), which indicated that spatial variation had a limited influence on the bacterial community structure (Supplementary Figure S4A). Conversely, bacterial community similarity exhibited a strong and significant relationship with physicochemical parameters (*r* = 0.65, *p* < 0.001). This suggested that the impact of physicochemical variables on the bacterial community was significantly stronger than geographic distance (Supplementary Figure S4B).

Supplementary Table S2 presented the physiochemical parameters at different seasons in the subsidence lake and natural river. The physicochemical parameters in the two seasons show significantly different. In particular, the concentrations of TN, BOD, NH_4_^+^ and ORP in winter were 2.46 ± 0.81 mg/l, 8.64 ± 1.52 mg/l, 0.85 ± 0.67 mg/l and 218 ± 5.67 mV, respectively, which were higher in summer (0.5 ± 0.18 mg/l, 6.2 ± 2.25 mg/l, 0.2 ± 0.12 mg/l, 130 ± 12.87 mV, respectively; *p* < 0.01). However, the *T* value and Chl-a and TP concentrations in winter (6.56 ± 0.37°C, 3.2 ± 1.85 mg/m^3^, and 0.84 ± 0.04 mg/l, respectively) were lower than in summer (20.36 ± 1.64°C, 8.34 ± 0.45 mg/m^3^, and 20.36 ± 1.52 mg/l, respectively; *p* < 0.01; Supplementary Table S2).

**Figure 4:**
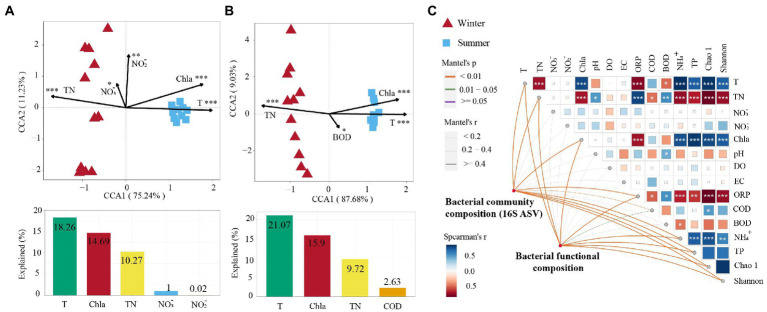
Drivers of bacterial community composition and bacterial function composition in subsidence lake and natural river. Canonical correspondence analysis (CCA) between **(A)** bacterial community composition and **(B)** function with significant environmental factors. Bar plots are shown below CCA presenting the variation explained by each factor. **(C)** The correlation of bacterial community composition, environmental factors, and bacterial functional composition using the Mantle test. ^*^p < 0.05, ^**^p < 0.01, ^***^p < 0.001.

The Mantel test found that bacterial community composition (BCC) was significantly correlated with T, TP, and NH_4_^+^ (*p* < 0.001; Figure 4C). The bacterial functional composition (BFC) was significantly correlated with T, Chl-a, and NH_4_^+^ (*p* < 0.001; Figure 4C). Moreover, correlation analysis found that the bacterial α-diversity indices (Chao 1 and Shannon) presented a significant positive correlation with T, Chl-a, and NH_4_^+^, while they presented a significant negative correlation with TN and ORP (*p* < 0.001; Figure 4C). CCA found that the variation of bacterial community structure was related to five environmental factors in the subsidence lake and natural river: T, Chl-a, TN, NO_3_^−^ and NO_2_^−^ (Figure 4A). These variables accounted for 44.24% of the variation in bacterial community structure. Among them, T (18.26%) was the most important factor in explaining the variation in bacterial community structure in seasonal changes (Figure 4A). The variation of bacterial community functional groups was significantly explained by four environmental parameters in the subsidence lake and natural river: T, Chl-a, TN and BOD (Figure 4B). These parameters accounted for 49.32% of the variation in bacterial community function. Among these factors, T (21.07%) was also the most crucial variable in accounting for the variation in BFC in seasonal changes (Figure 4B).

### Bacterial community assembly mechanisms between different seasons in subsidence lakes

**Figure 5:**
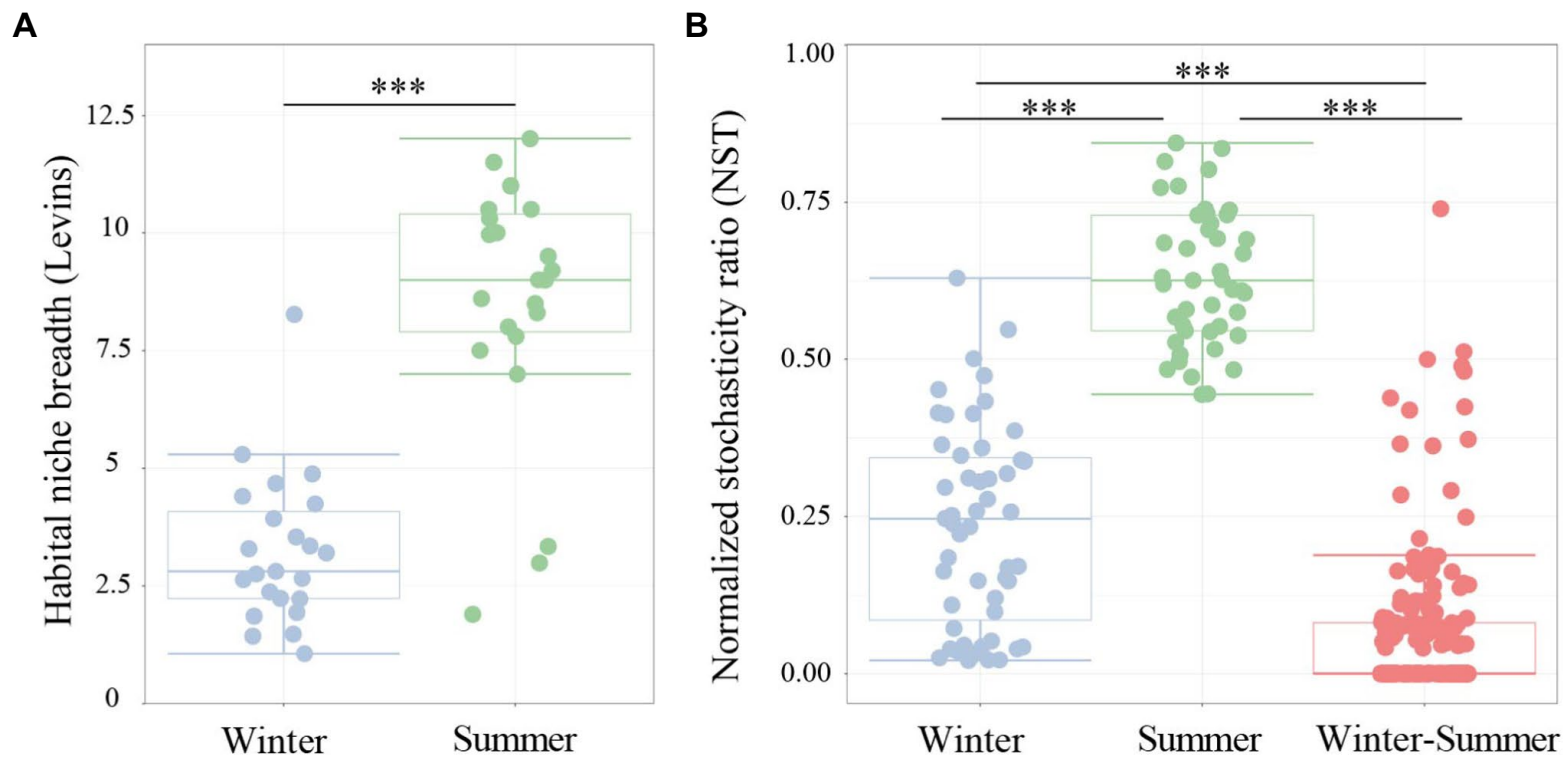
Boxplots presenting **(A)** the mean habitat niche breadth and **(B)** the ratio of stochasticity in bacterial community assembly from water samples of subsidence lake and natural river in different seasons. Two comparisons with Wilcoxon tests, ^*^p < 0.05, ^**^p < 0.01, ^***^p < 0.001.

To explore the assembly mechanism of microbial communities in subsidence lakes and natural rivers, the normalized stochasticity ratio (NST) was estimated. The findings showed that the relative contributions of deterministic (NST < 0.5) and stochastic (NST > 0.5) processes to aquatic bacterial community assembly were greatly influenced by climate change (Figure 5B). During winter, a lower value of NST (0.24 ± 0.18) was observed, which indicated that deterministic processes have a more crucial role in shaping winter bacterial community. Conversely, a higher relative contribution (NST = 0.63 ± 0.11) of stochastic processes was observed during summer (Figure 5B). In contrast, deterministic processes (NST = 0.06 ± 0.12) played a dominant role in explaining the variation in aquatic bacterial community structure during seasonal change (Figure 5B). Additionally, significantly higher habitat niche breadths were observed for summer (8.58 ± 2.61) than for winter (3.24 ± 1.55; Figure 5A; *p* < 0.001).

### Network analysis of bacterial communities

**Figure 6:**
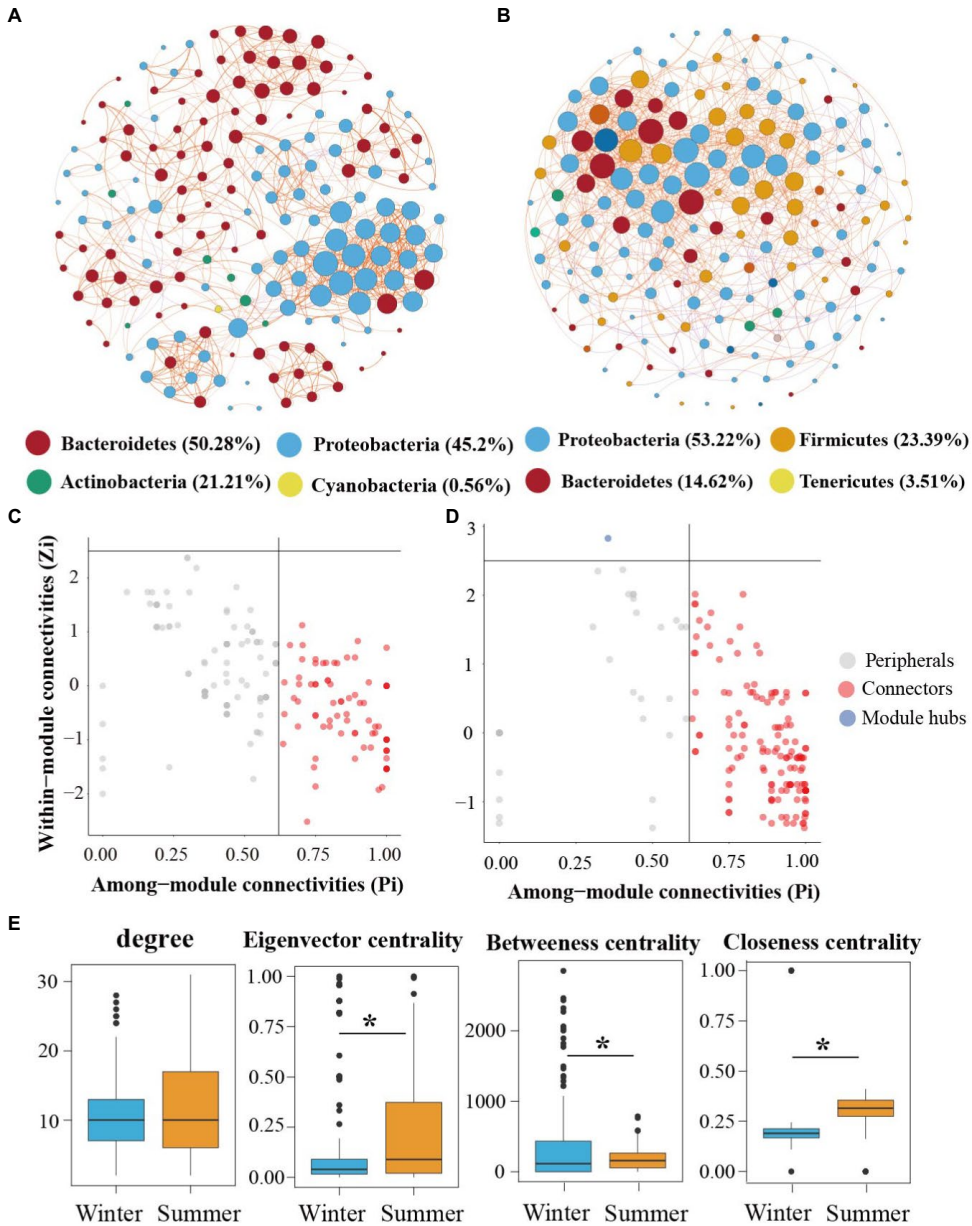
Network analysis of bacterial communities in subsidence lake and natural river. Networks analysis showing the bacterial community interspecies interactions in winter and summer **(A,B)**. The bacteria at the phylum level are colored by different color nodes. The size of the node presents the number of connections. The different color of edge color presents the positive (orange) and negative (purple) correlations of species. Identification of keystone taxa in **(C)** winter and **(D)** summer through their topological roles in networks. **(E)** The difference of topological features at node-level among two seasons network. Two comparisons with Wilcoxon tests, ^*^p < 0.05, ^**^p < 0.01, ^***^p < 0.001.

We further conducted the co-occurrence network to estimate the impact of seasonal variation on microbial communities co-occurrence patterns in the subsidence lake and natural river (Figures 6A,B). Based on network analysis, we recorded 177 nodes with 943 edges in winter and 171 nodes with 1,019 edges in summer bacterial community network (Supplementary Table S3). The network properties used to characterize bacterial community network stability (proportion of negative and modularity) increased from winter to summer (Supplementary Table S3). The coefficients (*σ*) of small-world > 1, indicate that the network had small-world properties (Supplementary Table S3).

The network of the bacterial community in winter (97.35%) and summer (90.58%) was mostly positively constructed, suggesting ecological mutualistic or cooperative relationships in the subsidence lake and natural river. The average degree and graph density were 11.92 and 0.07 in summer network, respectively, which were higher than in winter network, demonstrating the greater interactions and complexity of summer network (Supplementary Table S3). We further compared the specific topological features at the node level, including degree, closeness, betweenness, and eigenvector centrality, among the winter and summer (Figure 6E). Winter and summer networks have the same degree values. However, closeness centrality and eigenvector centrality values in winter were significantly higher (*p* < 0.001). However, a contrasting pattern was observed for betweenness centrality in the two different seasons (Figure 6E).

Most of the nodes in the two networks were mainly composed of *Proteobacteria*, *Bacteroidetes*, *Actinobacteria*, *Cyanobacteria*, *Firmicutes*, and *Tenericutes* (Figures 6A,B). Among them, in winter network, *Bacteroidetes* (50.28%) was the dominant phyla, while in summer network *Proteobacteria* (53.22%) was the dominant phyla. The putative keystone species were identified using the within-module connectivity (Zi) and among-module connectivity (Pi) values of taxa in the two-season network. Network hubs, connectors and module hubs were considered keystone species. The proportion and number of keystone species members increased with the season variation (Figures 6C,D). In winter, the main keystone species included *Flavobacterium*, *Acinetobacter*, *Pseudomonas*, and *Janthinobacterium*, while in summer, the main keystone species included *Bacteroides*, *Aeromonas*, *Lactobacillus*, *Cloacibacterium*, and *Dysgonomonas*.

## Discussion

### Variations in bacterial diversity between different seasons

In this study, we found that seasons, rather than habitats, perform a crucial role in determining the bacterial communities diversity in subsidence lakes and natural rivers (Figure 2A). In line with previous studies, we also observed the bacterial α-diversity was significantly higher in summer (Zhang et al., 2019a; Wang et al., 2022b; Figure 2A). Several factors could explain this observation. The warm environment was demonstrated to be beneficial for the growth of microbes in summer, increasing species diversity (Wang et al., 2022b). And the concentration of nutrients performs a crucial role in the variation in microbial diversity (Kolmonen et al., 2011). A previous study suggested that inorganic nitrogen (N) and phosphorus (P) are crucial for microbial growth (Hu et al., 2020), and maximum microbial diversity usually appears at intermediate levels of nutrients or productivity (Zwirglmaier et al., 2015). This notion is consistent with the finding that Chao 1 and Shannon indices present a significantly positive correlation with T, Chl-a, and NH_4_^+^, and the content of TP was significantly higher in summer (Figure 4C; Supplementary Table S2). In the subsidence lake ecosystem we explored, the eutrophication status in winter was depleted, while in summer, it was mesotrophic (Supplementary Table S2). This indicated that the abundance of nutrients in summer promotes bacterial growth and increases species diversity in the subsidence lake and natural river. Nevertheless, our findings were contrary to the results of Ren et al. (2019b), who investigated the seasonal variation in bacterial communities in Poyang Lake and found that bacterial communities in winter hold higher α-diversity than in summer. This discrepancy may be attributed to the complexity of hydrological characteristics. Poyang Lake had a complex hydrological characteristic in summer as it connected with the Yangtze River, which reduced the influence of nutrients on species diversity (Ren et al., 2019b).

In addition to community diversity, we also calculated the functional diversity in the subsidence lake and natural river within different seasons. The results showed higher functional diversity in summer (Supplementary Figure S2). This was consistent with the findings of studies on the spatial and temporal dynamics of the bacterial community in rivers (Wang et al., 2022b). This could a resistance mechanism of bacterial community function to respond low-temperature in winter (Mai et al., 2018). The functions associated with nitrate reduction and nitrate respiration were significantly enriched in summer. This result is in line with previous studies, which demonstrated that genes related to nitrate reduction had a higher abundance when nitrate was enriched (Yang et al., 2019). It appears to be an adaptive mechanism of the bacterial community in response to nutrient enrichment (Tang et al., 2021).

### Communities structure and assembly mechanisms of bacterial in different seasons

Linear regression analysis and PCoA results in this study showed that spatial differences had no significant impact on bacterial community structure, while seasonal changes had a significant impact on community structure (Figure 2B; Supplementary Figure S4). This was consistent with previous research results, which also found that geographical distance has no significant impact on bacterial community similarity (Tang et al., 2020; Shen et al., 2022; Zhang et al., 2023). This may be due to the fact that the subsidence lake in this study was an open lake, which can exchange water with rivers frequently. The hydrodynamic effect of rivers caused the homogenization of lake water quality (Zhu et al., 2019), so the difference of bacterial community in different spaces was not significant. The significant difference of bacterial community in different seasons may be caused by the significant difference of physical and chemical indicators in different seasons. This is supported by the results that physical and chemical indicators have a significant impact on community similarity (Supplementary Figure S4b). In winter, *Flavobacterium* (58.4%) and *Acinetobacter* (15.4%) were significantly enriched (Figure 3B). Both *Flavobacterium* and *Acinetobacter* were cold-tolerant microorganisms. *Flavobacterium* could secrete cryoprotective extracellular polymeric substances, enabling them to be enriched in low-temperature environment (Zhang et al., 2020). It was found that the membrane transport protein and unsaturated fatty acid dehydrogenase-related genes of *Acinetobacter* were significantly upregulated at 5°C, enabling it to survive at low temperatures (Zhao et al., 2021). Moreover, *Flavobacterium* and *Acinetobacter* had high metabolic capacity for nitrogen (ammonia nitrogen, nitrate, etc.) at low temperature (Zhao et al., 2021; Zheng et al., 2021). This may be due to the environmental pressure caused by low temperature and high total nitrogen in winter driving the significant enrichment of *Flavobacterium* and *Acinetobacter*. On the contrary, *Bacteroides* (13.9%), and *Lactococcus* (12.4%) were significantly enriched in summer (Figure 3B). It is found that *Bacteroides* could effectively obtain organics from algae and their abundance and distribution are directly related to algae in the water environment (Shen et al., 2020). This was consistent with the high content of Chl-a in summer in this study (Supplementary Table S2). This may be due to the increase of temperature during summer, which causes the multiplication of algae, thus causing the significant enrichment of *Bacteroides*. *Lactococcus* was a pathogenic bacterium in commercial fish, and its activity was strong when the water temperature was high (Soltani et al., 2008). This may be due to the more frequent fish farming activities and the increased of temperature, which promote the activity of *Lactococcus* and made it significantly enriched in summer.

The mechanisms and processes driving bacterial community diversity and distribution patterns were further examined in subsidence lakes. A novel finding of this study is that the deterministic process was the main assembly mechanism impacting the community variation in winter, whereas stochastic processes had a higher impact in summer (Figure 5B). In winter, TN may be one of the crucial deterministic processes in subsidence lakes. This was supported by the fact that TN explained 25.5% of the total community variations in summer solely (Supplementary Figure S5a). In the high-TN aquatic habitat, the strong selection pressure exerted by the increased TN content will perform a stringent filter on microbial communities, and only the dominant community adapted to this high TN ecological niche will proliferate (Craig et al., 2021). This was tied well with the present study, and we found that the dominant genus in the subsidence lake during winter was mainly involved in the metabolism of ammonia nitrogen. For instance, the dominant genera *Flavobacterium* (58.4%) and *Acinetobacter* (15.4%) have a high ability to assimilate and metabolize ammonia nitrogen under low-temperature conditions (Zhao et al., 2021; Zheng et al., 2021). Additionally, higher environmental heterogeneity in the subsidence lake during winter may provide more ecological niches for bacterial communities, thus increasing the relative significance of deterministic processes (Tang et al., 2021). Similar to other lake ecosystems, the study found that deterministic mechanisms such as environmental filtering performed a crucial role in controlling the bacterial community variation between the winter and summer (Figure 5B). Among the winter and summer, bacterial community similarity exhibited a strong and significant relationship with physicochemical parameters (*r* = 0.65, *p* < 0.001; Supplementary Figure S4), and T explained 18.26% of the total community variations solely (Figure 4A), which is evidence for environmental filtering. The environmental stress on the temperature may boost the filtering out of communities with weak cold resistance and narrow niche breadth in winter (He et al., 2022). However, warm weather conditions were conducive to the growth of species and the maintenance of biodiversity (Wang et al., 2022b). Therefore, the temperature was one of the main deterministic processes impacting the community variation between the winter and summer in subsidence lakes.

In the subsidence lake during summer, the contribution of the deterministic mechanisms on impacting the bacterial community was small, demonstrating that the stochastic mechanisms were more crucial (Figure 5B). This was consistent with what had been found in previous plateau lakes, which found that the increased temperature in summer promoted microbial metabolic rates, thereby increasing the randomness of speciation and death (He et al., 2022). Concurrently, the increasing relative importance of stochastic processes in summer was well associated with higher nutrient contents. With the increase in T and nutrients, the dominant community in winter could lose its growth advantage. In summer, microbial communities that have unique physiological responses to increases in temperature and nutrients will have sufficient nutrients for growth, without having to oppose the pressure of cold temperatures (She et al., 2021). The large proliferation of microbial communities could strengthen stochastic processes (birth, death, etc.), which in turn cause stochastic variation in microbial communities abundance (Zhou et al., 2014). The large proliferation of microbial communities further causes a considerable increase in microbial diversity during summer, unlike the typical dominant communities during winter (Figure 3). This was consistent with the result that in summer species and functional diversity were higher than those in winter (Figure 2). Additionally, summer with suitable temperatures and abundant resources could decrease ecological niche selection. In summer, we observed a broader niche breadth, which demonstrated the microbial community metabolism was more flexible (Figure 5A; Wu et al., 2017; Jiao et al., 2020b). The different controlling mechanisms between different seasons of microbial community assembly found in the study have been observed in other aquatic ecosystems, such as plateau lakes (He et al., 2022) and water diversion canals (Zhang et al., 2021). Therefore, our comparative analyses between different seasons of the bacterial community provide the first informative insights into the spatial- and temporal-dependent patterns in subsidence lake community assembly.

### Temporal dynamics of bacterial communities co-occurrence patterns in subsidence lakes

Network analysis further presents us with a comprehensive better comprehension of the community assembly mechanisms across different seasons in coal mining subsidence lakes. Our study showed that in summer the network with the largest edge exhibited more complex interactions among the ASVs than in winter, as supported by the topological features in node- and network-level (Figures 6A,E; Supplementary Table S3). This result is consistent with research on planktonic bacterial communities in lakes which found that the co-occurrence networks developed from complex to simple from summer to winter (Jiao et al., 2020a). Nevertheless, our findings were contrary to the results of Shi et al., who found that the network connectivity and complexity of the epiphytic bacterial community gradually increased from July to November (Shi et al., 2022). This discrepancy may be attributed to the difference in bacterial diversity in different seasons: higher diversity in summer in this study and higher diversity in November in the study by Shi. Previous studies found that higher microbial diversity could promote species interactions, thus increasing the complexity of the co-occurrence network (Widder et al., 2014; Sun et al., 2021). In the subsidence lake, network modularity was higher in summer, indicating that the bacterial community in summer could be more stable since the high modularity of the network restricts the influence of species loss on the overall community (Hernandez et al., 2021). Additionally, we observed more positive edges in the bacterial network during winter, which suggests bacterial communities in winter had a higher niche overlap and positive interspecific interactions. In line with previous studies, microbes could use limited resources *via* efficient cooperation when nutrients were depleted in winter (Dang et al., 2015). However, such positive interactions may destroy the stability of the microbial community, as species that rely on feedback loops may disintegrate from each other (Coyte et al., 2015; Stone, 2020). In addition, the average path length and average clustering coefficient were lower in summer, indicating a more rapid transfer of information and materials and more frequent ecological linkages and communication among different species in summer. The differences of bacterial community complexity and stability between seasons in subsidence lakes may be due to the temperature variation. The temperature was supposed to the essential elements impacting seasonal variation in microbial co-occurrence relationships (Ren et al., 2019a). Temperature could impacts other water environmental variables (DO, Chl-a, etc.) simultaneously, thereby strongly influencing microbial diversity and co-occurrence patterns in lake ecosystems (Wang et al., 2019).

Keystone species perform a crucial role in sustaining the complexity and stability of the microbial community network, and their loss may cause the disintegration of the community structure (Banerjee et al., 2018; Xue et al., 2018). Many studies have focused on dominant species in microbial communities, as well as specific species that play crucial roles in community structure and function (Zhou et al., 2022a). Based on network scores (Pi and Zi), we predicted the potential keystone taxa among different seasons in coal mining subsidence lakes (Figure 6 cd). In the present study, results indicate that summer network includes more keystone species, indicating that a microbial community including more keystone species could accelerate the stability of microbial communities. This is consistent with the results of She et al., who found that keystone species in acid mine drainage lakes can enhance the complexity and stability of the microbial community (She et al., 2021). In addition, seasonal variations can alter keystone species in a specific environment, so keystone species may only exist in a specific season (Liu et al., 2022). In our study, the main keystone species in winter included *Flavobacterium*, *Acinetobacter*, *Pseudomonas*, and *Janthinobacterium*, while in summer, the main keystone species included *Bacteroides*, *Aeromonas*, *Lactobacillus*, *Cloacibacterium*, and *Dysgonomonas*. Those keystone species in winter were all dominant genera, while in summer, they were partially rare. This indicated that winter with high TN and low T formed an environmental filter that retained a high abundance of key functional bacteria to form an effective functional microbial community. This is important for the effective resistance of microbial communities to extreme environments. The abundant nutrients and suitable temperature during summer may have promoted the formation of a large number of low-abundance functional bacteria, thus enhancing community diversity and stability. For example, *Flavobacterium* and *Acinetobacter* have a high ability to assimilate and metabolize ammonia nitrogen under low temperature conditions (Zhao et al., 2021; Zheng et al., 2021), and *Pseudomonas* and *Janthinobacterium* are good fixers and degraders of lignin and glucose in aquatic and soil ecosystems (Wilhelm et al., 2019; Kumar et al., 2021). In summer, *Bacteroides* is an important indicator species of fecal pollution in water (Gómez-Doñate et al., 2016), and *Aeromonas* is highly resistant to antibiotics (Skwor et al., 2014). *Cloacibacterium* has considerable metabolic diversity, which is participated in the disintegration of complex organic matter and has a removal effect on phosphates and heavy metals (Allen et al., 2006; Nouha et al., 2016; Kopprio et al., 2020). Overall, these keystone species perform an indispensable role in the carbon, nitrogen and phosphorus cycles and are of ecological significance in maintaining the seasonal stability of aquatic ecosystems in subsidence lakes. Additionally, our results on the keystone species are consistent with Zeng et al. (2021), who found that 9% of OTUs in artificial wetlands were identified as central species. Conversely, our results contrast with the findings of other inland lakes, in which only few keystone species were found in the different seasons of the lake (Jiao et al., 2020a; Liu et al., 2022). This difference may be due to the long-term influence of various natural and anthropogenic factors on coal mining subsidence lakes, such as the increasing depth and area of the lakes caused by underground coal mining, high-density seine farming, and the release of various elements into the lakes by the weathering of coal gangue. Therefore, a large number of keystone species in the microbial network of subsidence lakes can resist various environmental perturbations to maintain community stability.

## Conclusion

This study illustrates the distinct seasonal heterogeneity of the bacterial community in subsidence lakes with strong natural and anthropogenic disturbances and demonstrates the underlying community assembly mechanisms in different seasons, providing a valuable reference for the management and efficient utilization of water resources in mining areas. Here, we showed that seasonal variation performs a significant impact on the bacterial communities structure and diversity in subsidence lakes, and the α-diversity and functional diversity of bacterial communities were highest in summer. In addition, we found that deterministic processes dominated community assembly in winter, while stochastic processes were the leading mechanism controlling the community assembly in summer. T and Chl-a were the main deterministic process in impacting the seasonal variation of bacterial communities in subsidence lakes. In summer, the bacterial community network had a higher average degree, modularity, and keystone taxa (hubs and connectors in a network), implying that the community structure was more complex and stable. In this study, microbial assemblage patterns between winter and summer were analyzed. In the future, a broader spatial and temporal scale can be considered to explore the variation of microbial community diversity and assembly processes in subsidence lakes.

## Data availability statement

The datasets presented in this study can be found in online repositories. The names of the repository/repositories and accession number(s) can be found at: https://www.ncbi.nlm.nih.gov/, PRJNA923449.

## Author contributions

WF: writing—original draft. TF: conceptualization and funding acquisition. LX: formal analysis. SW: investigation. XW: software and investigation. AL: supervision and validation. YC: investigation and resources. All authors contributed to the article and approved the submitted version.

## Funding

This work was supported by the National Natural Science Foundation of China (51878004) and the Research Foundation of Huaibei Mining Group in 2021, Research Foundation of Huainan Mining Group in 2021, China Energy Investment Corporation 2030 Pilot Project (grant numbers GJNY2030XDXM-19-03.2), Research Foundation of Huaibei Mining Group in 2022, and Research Foundation of the Institute of Environment-friendly Materials and Occupational Health (Wuhu), Anhui University of Science and Technology (ALW2020YF08).

## Conflict of interest

The authors declare that the research was conducted in the absence of any commercial or financial relationships that could be construed as a potential conflict of interest.

## Publisher’s note

All claims expressed in this article are solely those of the authors and do not necessarily represent those of their affiliated organizations, or those of the publisher, the editors and the reviewers. Any product that may be evaluated in this article, or claim that may be made by its manufacturer, is not guaranteed or endorsed by the publisher.
