# Supplementary material for: Seasonal succession of microbial community co-occurrence patterns and community assembly mechanism in coal mining subsidence lakes
Source: Front Microbiol. 2023 Feb 3;14:1098236. doi: 10.3389/fmicb.2023.1098236 (PMC9936157; doi:10.3389/fmicb.2023.1098236)

**TABLE S1** Diversity index and amplicon sequence variants (ASVs) of water samples in subsidence lakes and natural rivers.

|  | Samples | ASV | Chao 1 | Shannon | Coverage |
| --- | --- | --- | --- | --- | --- |
| Winter | R1 | 463 | 397.31 | 2.62 | 0.996 |
|  | R2 | 389 | 595.91 | 3.80 | 0.997 |
|  | R3 | 557 | 605.13 | 3.55 | 0.996 |
|  | R4 | 1712 | 377.03 | 1.77 | 0.997 |
|  | R5 | 576 | 521.61 | 2.91 | 0.996 |
|  | L1 | 332 | 531.45 | 2.68 | 0.997 |
|  | L2 | 533 | 433.89 | 2.38 | 0.996 |
|  | L3 | 534 | 597.50 | 3.45 | 0.996 |
|  | L4 | 348 | 1756.54 | 5.21 | 0.997 |
|  | L5 | 468 | 637.61 | 3.24 | 0.996 |
|  | R6 | 618 | 680.81 | 3.46 | 0.996 |
| Summer | R1 | 2721 | 3200.26 | 5.02 | 0.976 |
|  | R2 | 2688 | 3680.22 | 5.15 | 0.976 |
|  | R3 | 2354 | 3332.41 | 5.01 | 0.981 |
|  | R4 | 2756 | 3293.66 | 5.15 | 0.976 |
|  | L1 | 2314 | 3687.52 | 5.27 | 0.981 |
|  | L2 | 2716 | 3781.24 | 5.13 | 0.977 |
|  | L3 | 2358 | 3656.05 | 5.04 | 0.980 |
|  | L4 | 2282 | 3243.61 | 5.03 | 0.981 |
|  | L5 | 2528 | 3715.42 | 5.14 | 0.978 |
|  | R6 | 2866 | 3872.27 | 5.10 | 0.974 |

**TABLE S2** Physicochemical parameters of samples in subsidence lakes and natural rivers. * *P* < 0.05, ** *P* < 0.01, *** *P* < 0.001.

|  | Samples | T^***^  ℃ | TN^***^  mg/L | NO_3_^-^  mg/L | NO_2_^-^  mg/L | Chla^***^  mg/m^3^ | pH | DO  mg/L | EC  us/cm | ORP^***^  mv | COD  mg/L | BOD^**^  mg/L | NH_4_^+***^  mg/L | TP^***^  mg/L |
| --- | --- | --- | --- | --- | --- | --- | --- | --- | --- | --- | --- | --- | --- | --- |
| Winter | R1 | 6.5 | 1.52 | 0.25 | 0.03 | 1.83 | 8.57 | 12.3 | 861 | 221 | 44 | 9 | 0.70 | 0.05 |
|  | R2 | 6.6 | 2.85 | 0.15 | 0.03 | 1.56 | 8.29 | 9.2 | 935 | 223 | 39 | 7.8 | 0.59 | 0.06 |
|  | R3 | 6.8 | 3.21 | 0.06 | 0.09 | 1.96 | 8.85 | 13.8 | 900 | 210 | 60 | 7.9 | 0.18 | 0.11 |
|  | R4 | 6.9 | 1.38 | 0.23 | 0.32 | 7.31 | 8.58 | 9.30 | 948 | 212 | 112 | 7.3 | 0.59 | 0.13 |
|  | R5 | 7.2 | 1.67 | 0.37 | 0.06 | 3.04 | 8.45 | 10.9 | 1034 | 213 | 181 | 8.1 | 2.84 | 0.11 |
|  | L1 | 5.8 | 2.05 | 0.20 | 0.04 | 2.98 | 8.89 | 13.8 | 928 | 224 | 49 | 7.3 | 1.16 | 0.16 |
|  | L2 | 6.6 | 3.21 | 1.21 | 0.36 | 6.26 | 8.89 | 8.30 | 920 | 223 | 15 | 9.08 | 0.57 | 0.15 |
|  | L3 | 6.7 | 2.17 | 0.34 | 0.41 | 4.15 | 8.78 | 9.10 | 971 | 225 | 16 | 10 | 0.85 | 0.15 |
|  | L4 | 6.5 | 3.37 | 0.23 | 0.04 | 2.78 | 8.58 | 9.40 | 997 | 224 | 15 | 8.7 | 0.58 | 0.17 |
|  | L5 | 6.6 | 3.82 | 0.11 | 0.05 | 1.82 | 8.84 | 10.18 | 924 | 213 | 20 | 12.9 | 0.75 | 0.14 |
|  | R6 | 6 | 1.85 | 0.17 | 0.05 | 1.63 | 8.45 | 5.80 | 1056 | 213 | 99 | 8.2 | 0.49 | 0.08 |
| Summer | R1 | 20.5 | 0.53 | 0.10 | 0.27 | 18.74 | 8.38 | 10.9 | 987 | 120 | 71.8 | 9.7 | 0.36 | 10.9 |
|  | R2 | 20.5 | 0.28 | 0.07 | 0.03 | 3.32 | 7.4 | 11.5 | 1231 | 140 | 64.2 | 6.3 | 0.05 | 11.5 |
|  | R3 | 23 | 0.72 | 0.72 | 0.03 | 6.13 | 7.63 | 6.5 | 1252 | 140 | 95.4 | 1.9 | 0.11 | 6.5 |
|  | R4 | 21.9 | 0.63 | 0.14 | 0.05 | 5.98 | 8.53 | 9.1 | 820 | 138 | 29.4 | 3.1 | 0.09 | 9.1 |
|  | L1 | 19.3 | 0.91 | 0.43 | 0.03 | 11.91 | 8.82 | 12.2 | 953 | 147 | 59 | 8.5 | 0.09 | 12.2 |
|  | L2 | 19.1 | 0.32 | 0.37 | 0.30 | 15.88 | 8.36 | 10.7 | 953 | 145 | 59 | 7.1 | 0.29 | 10.7 |
|  | L3 | 18.5 | 0.56 | 0.30 | 0.32 | 16.45 | 8.75 | 9.5 | 1014 | 120 | 70.2 | 5.8 | 0.33 | 9.5 |
|  | L4 | 19.2 | 0.44 | 0.20 | 0.04 | 25.29 | 8.66 | 11.3 | 956 | 116 | 68.2 | 8.2 | 0.33 | 11.3 |
|  | L5 | 18.6 | 0.54 | 0.18 | 0.17 | 18.23 | 8.33 | 10.1 | 919 | 116 | 67 | 6.5 | 0.25 | 10.1 |
|  | R6 | 23 | 0.44 | 0.17 | 0.16 | 19.48 | 8.70 | 10.1 | 831 | 113 | 75.8 | 6.6 | 0.30 | 10.1 |

**TABLE S3** Topological properties of bacterial co-occurrence networks in subsidence lakes and natural rivers.

| Empirical network | | | | | | | | | | |  | Random network | | |
| --- | --- | --- | --- | --- | --- | --- | --- | --- | --- | --- | --- | --- | --- | --- |
|  | N | E | | Modularity | avgCC | APL | ND | AD | GD | σ |  | Modularity | avgCC | APL |
|  |  | Positive | Negative |  |  |  |  |  |  |  |  |  |  |  |
| Winter | 177 | 918 (97.35%) | 25 (2.65%) | 0.35 | 0.794 | 5.59 | 15 | 10.66 | 0.061 | 4.89 |  | 0.24 | 0.078 | 2.7 |
| Summer | 171 | 923 (90.58%) | 96 (9.42%) | 0.62 | 0.566 | 3.31 | 9 | 11.92 | 0.07 | 3.39 |  | 0.12 | 0.134 | 2.65 |

N: Nodes; E: Edges; avgCC: Average clustering coefficient; APL: Average path length; ND: network diameter; AD: Average degree; GD: Graph density; σ: small-word coefficient.

σ = (avgCC/avgCC*r*)/(APL/APL*r*) and σ > 1 indicates “small-world” properties.

**TABLE S4** Keystone species of the winter and summer network in subsidence lakes.

| Season | Keystone genus | Number of ASVs | Season | Keystone genus | Number of ASVs |
| --- | --- | --- | --- | --- | --- |
| Winter | *Acinetobacter* | 17 | Summer | *Bacteroides* | 26 |
|  | *Flavobacterium* | 20 |  | *Aeromonas* | 19 |
|  | *Pseudomonas* | 6 |  | *Lactobacillus* | 13 |
|  | *Janthinobacterium* | 6 |  | *Cloacibacterium* | 14 |
|  | *Massilia* | 7 |  | *Dysgonomonas* | 10 |
|  | *Algoriella* | 5 |  | *Neisseria* | 11 |
|  | *Delftia* | 2 |  | *hgcI_clade* | 6 |
|  | - | - |  | *Roseomonas* | 8 |
|  | - | - |  | *Mitochondria* | 5 |
|  | - | - |  | *Arcobacter* | 5 |
|  | - | - |  | *Muribaculaceae* | 1 |
|  | - | - |  | *Enterococcus* | 1 |
|  | - | - |  | *Shewanella* | 1 |
|  | - | - |  | *Thiothrix* | 1 |
|  | - | - |  | *Macrococcus* | 1 |

**FIGURE S1** Rarefaction curves of richness of water samples in subsidence lakes and natural rivers. **(a)** winter; (**b)** summer.

**FIGURE S2** Heatmap representing the abundance of predicted functions among different seasons based on FAPROTAX. **(a)** winter; (**b)** summer.

**FIGURE S3** Functional difference between different seasons.

**FIGURE S4** Linear regression analysis between the Bray–Curtis similarity of bacterial communities, geographical distance and physicochemical parameters in subsidence lake and natural river. (a) (b) all samples; (c) (d) winter; (e) (f) summer.

**FIGURE S5** Canonical correspondence analysis (CCA) between bacterial community and environmental factors. **(a)** winter; (**b)** summer.

**FIGURE S6** Co-occurrence network analysis of bacterial communities in subsidence lakes and natural rivers. **(a)** winter; (**b)** summer.

**FIGURE S1**


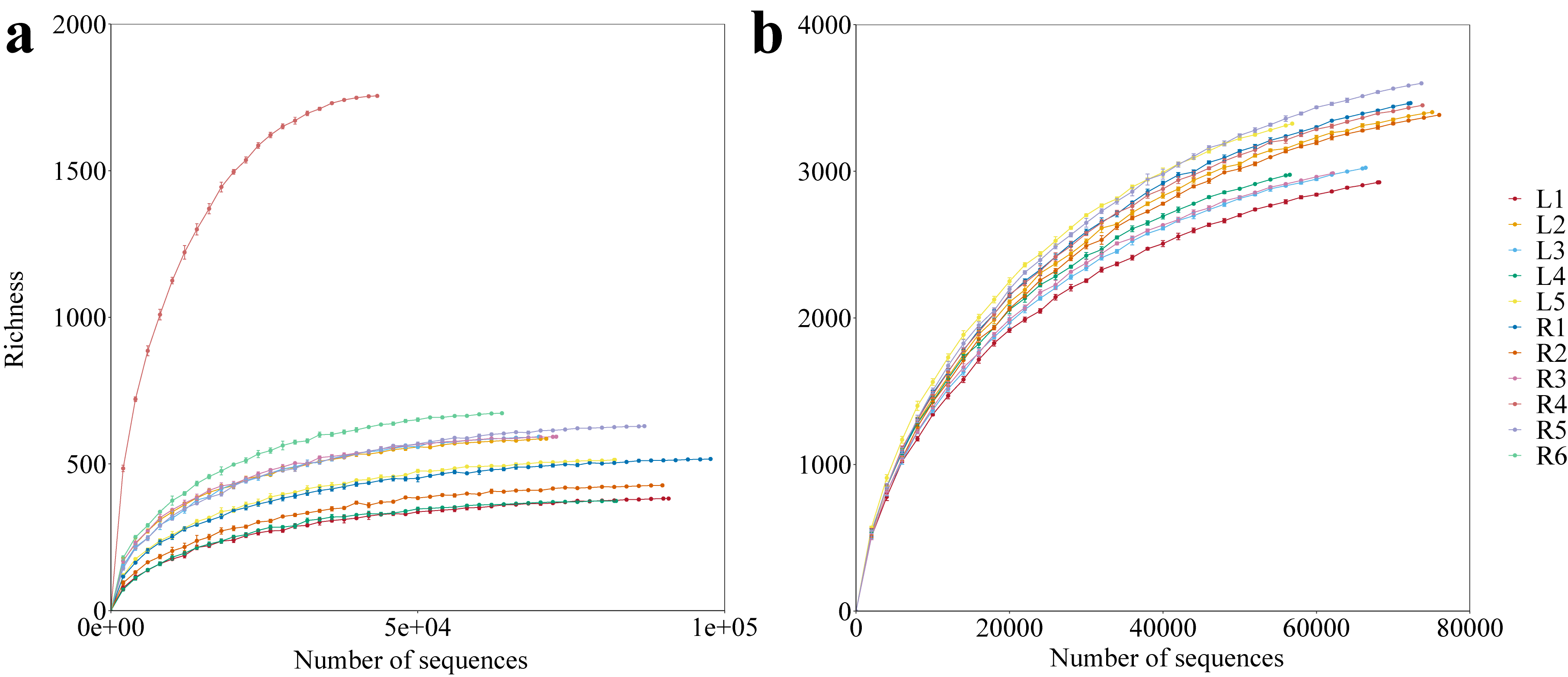


**FIGURE S2**

**
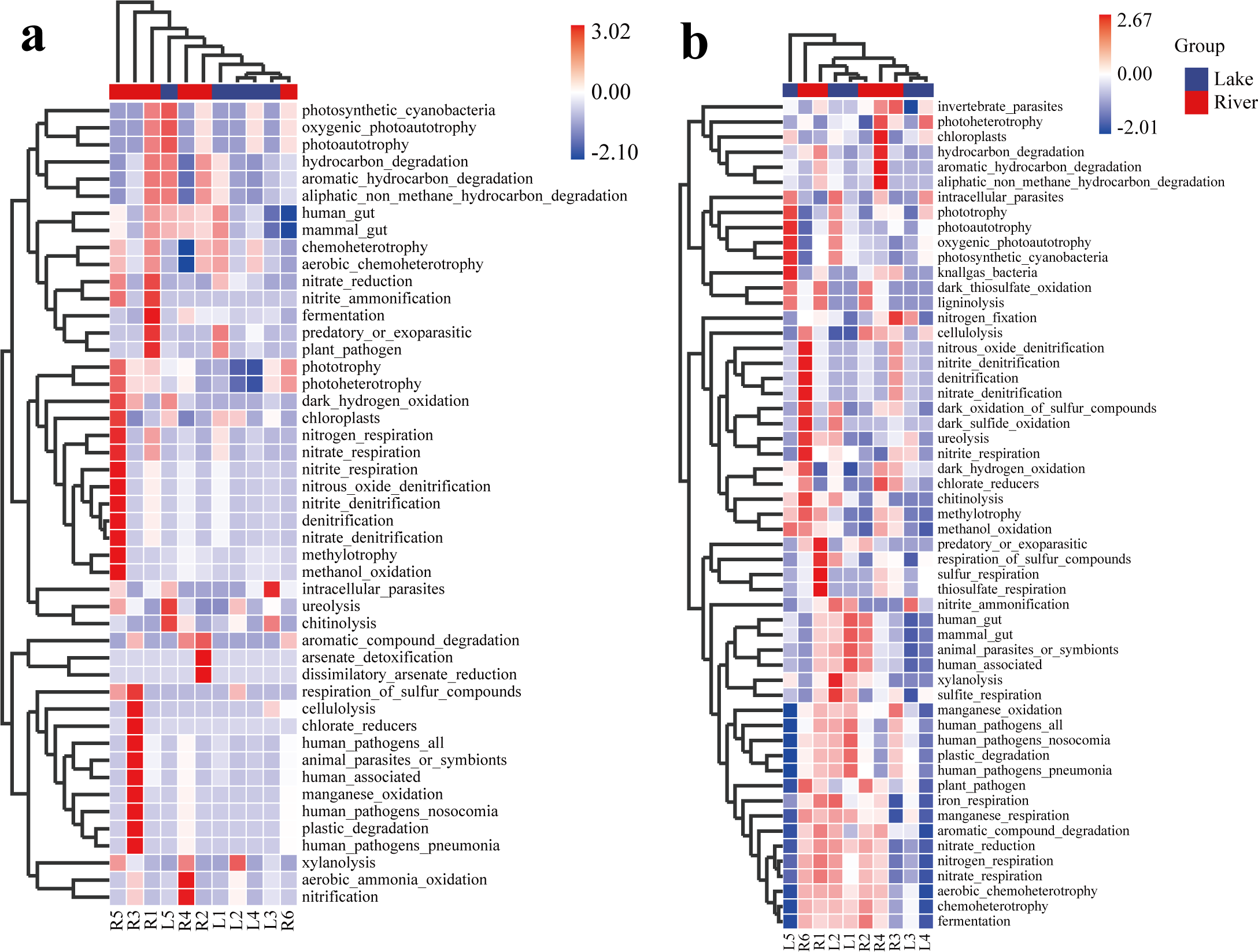
**

**FIGURE S3**

**
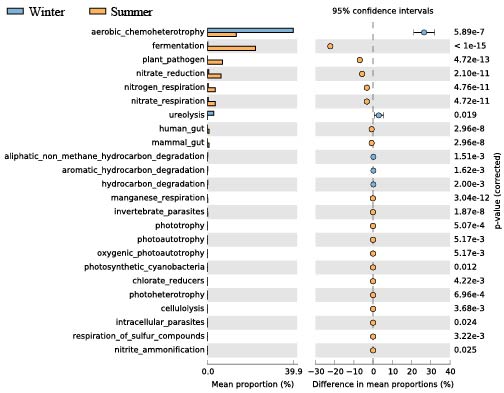
**

**FIGURE S4**

**
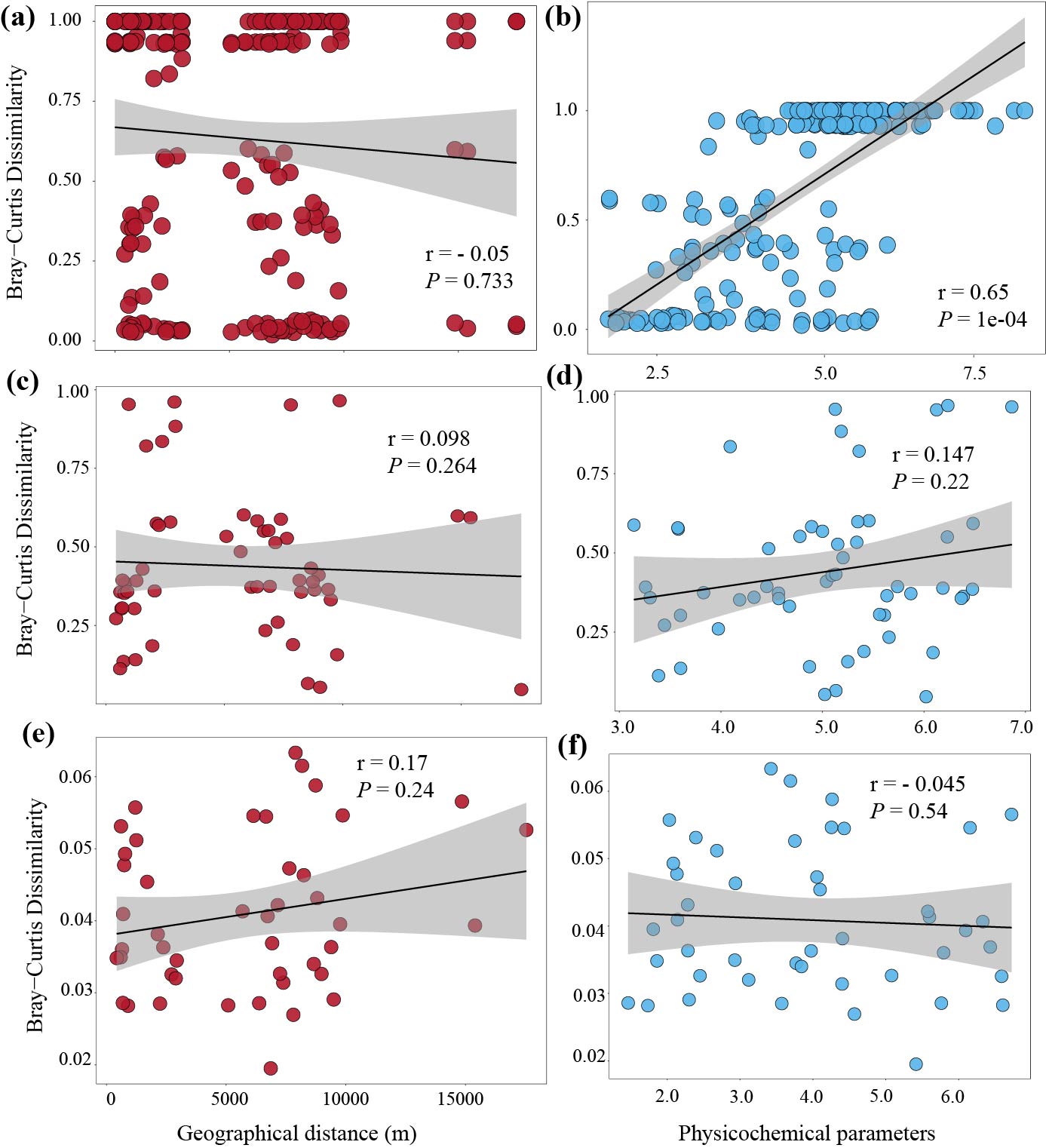
**

**FIGURE S5**

**
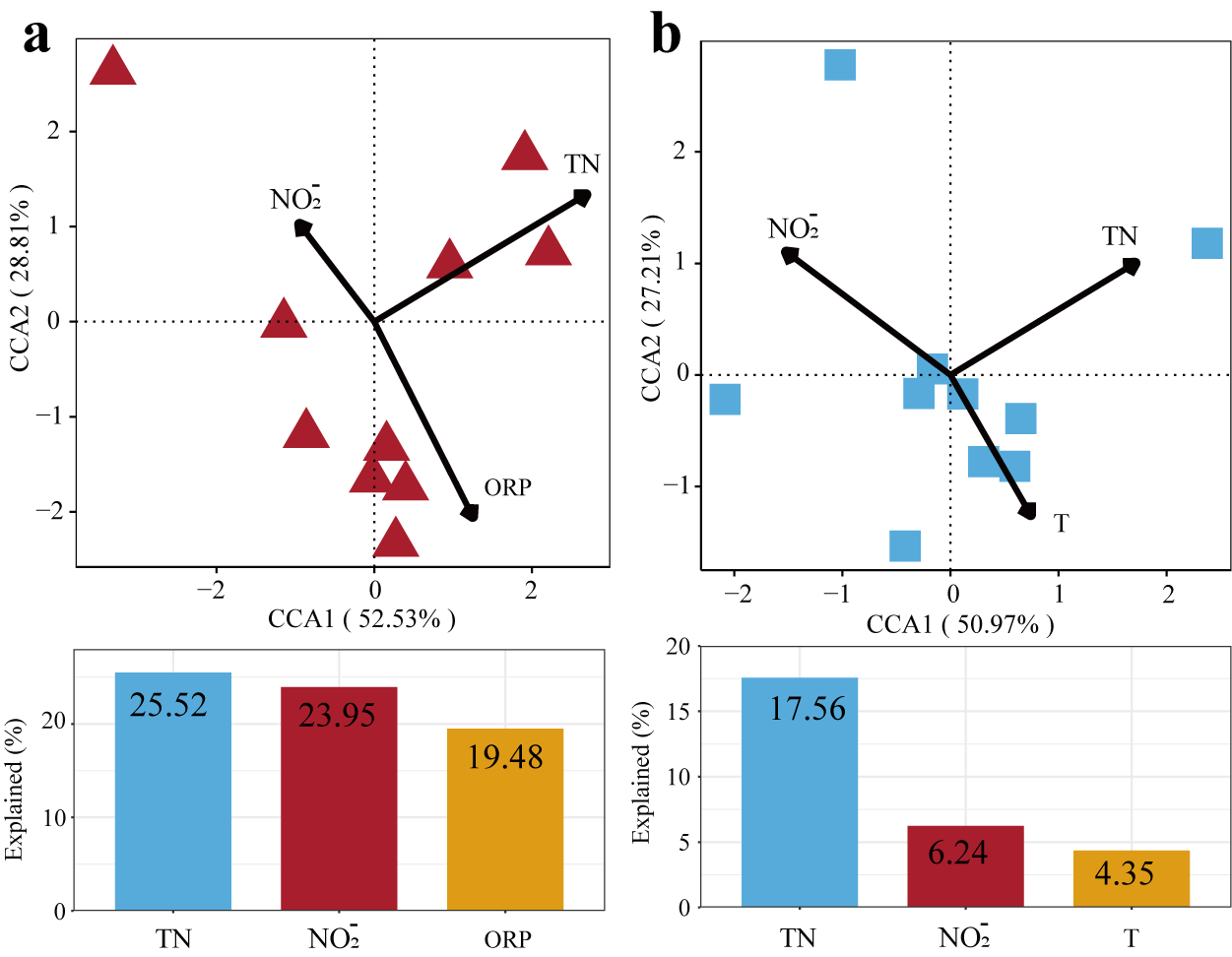
**

**FIGURE S6**


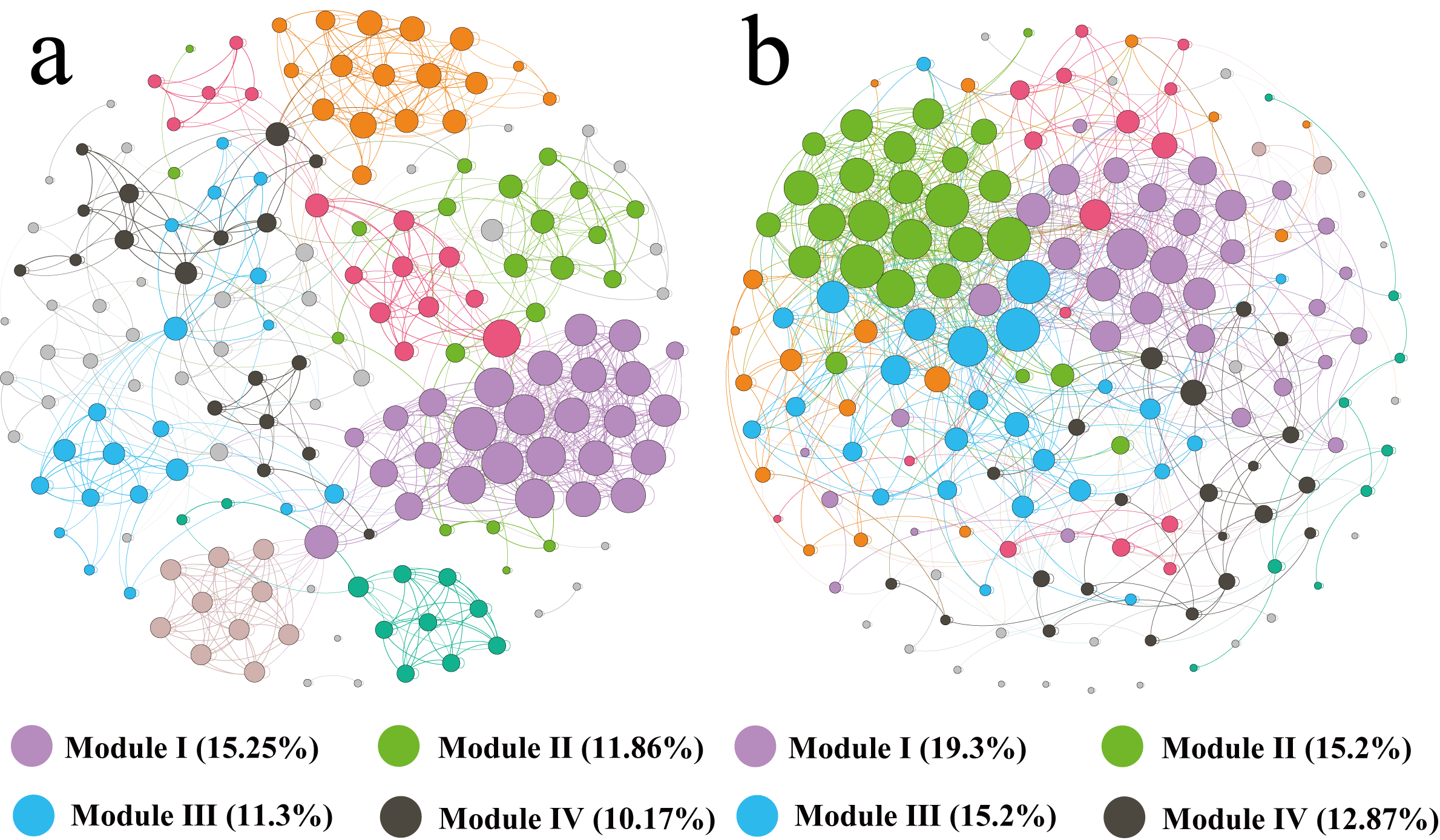

Supplement: Supplementary file 1 [file Table_1.docx]
